# La cyclopie: malformation rare du visage dans un centre de santé de référence de Bamako à propos d'un cas

**DOI:** 10.11604/pamj.2019.33.261.19240

**Published:** 2019-07-29

**Authors:** Doumbia Amadou, Koné Youssouf

**Affiliations:** 1 Service de Radiologie du Centre de Santé de Référence de la Commune VI de Bamako, Mali; 2 Service de Radiologie du Centre Hospitalier Jacques Boutard, Saint-Yrieix-la-Perche, Franc

**Keywords:** Cyclopie, malformation, bourgeon frontal, Cycolopia, malformation, frontal bud

## Abstract

Cyclopia also called “cyclopia” is a rare genetic malformation. It is the most severe form of alobaric holoprosencephaly. It is a rare form of hypotelorism characterized by the fusion of the two orbits and the presence of a single eye in the middle of the forehead hence its name. This malformation is associated with other facial malformations. Cyclopia is related to the absence of development of the frontal bud returning within the scope of ectroprosopia. ormation, face. We report a case of cyclopia in a newborn female born from a pregnancy estimated at term. The delivery was dystocic and the mother did not attend any prenatal consultations. The interrogation does not find any bond of consanguinity of the parents of the newborn. Absence of malformation in the siblings which is composed of four hildrens. At birth, the newborn weighed 4600 grams with a height of 58 cm. The examination of the newborn revealed a malformation of the face with a large single eye in the middle of the forehead and a total absence of the nose and orbits evoking the diagnosis of a cyclopia. The newborn died about ten minutes after his birth. He stayed alive just a few minutes No further exploration for etiological purposes could be carried out because of the parents' refusal.

## Image en médecine

La cyclopie également appelée «cyclope» est une malformation génétique rare. Elle est la forme la plus sévère de l'holoprosencéphalie alobaire. Il s'agit d'une forme rare de l'hypotélorisme se caractérisant par la fusion des deux orbites et la présence d'un seul œil au milieu du front d'où son nom. Cette malformation est associée à d'autres malformations du visage. La cyclopie est liée à l'absence de développement du bourgeon frontal rentrant dans le cadre de l'ectroprosopie. Nous rapportons un cas de cyclopie chez un nouveau-né de sexe féminin issu d'une grossesse estimée à terme. L'accouchement était dystocique et la mère n'a suivi aucune consultation prénatale. L'interrogatoire ne retrouve pas de lien de consanguinité des parents du nouveau-né. Absence de malformation dans la fratrie qui est composée de 4 enfants. À la naissance, le nouveau-né pesait 4600 grammes avec une taille de 58 cm. L'examen du nouveau-né mettait en évidence une malformation du visage avec un gros œil unique au milieu du front et une absence totale du nez et des orbites évoquant le diagnostic d'une cyclopie. Le nouveau-né est décédé une dizaine de minutes après sa naissance. Aucune exploration complémentaire à visée étiologique n'a pu être réalisée à cause du refus des parents.

**Figure 1:**
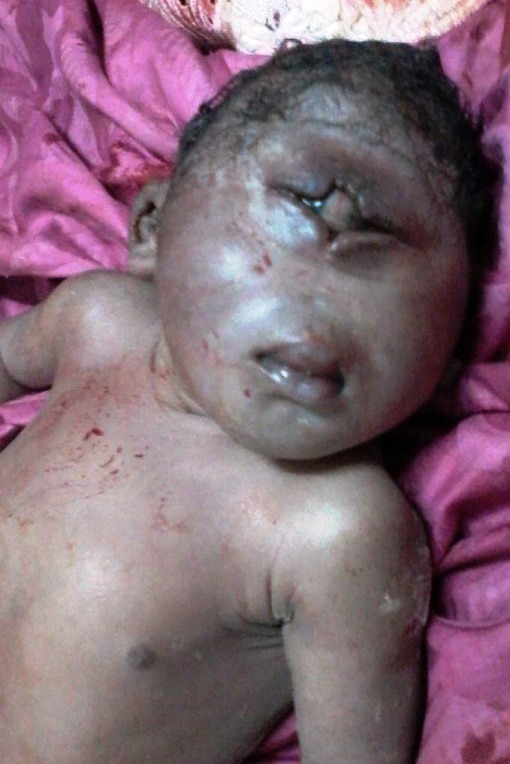
Malformation du visage avec un gros oeil unique au milieu du front et une absence totale du nez et des orbites

